# Developmental Toxicity of Mycotoxin *Fumonisin* B_1_ in Animal Embryogenesis: An Overview

**DOI:** 10.3390/toxins11020114

**Published:** 2019-02-13

**Authors:** Chompunut Lumsangkul, Hsin-I Chiang, Neng-Wen Lo, Yang-Kwang Fan, Jyh-Cherng Ju

**Affiliations:** 1 Department of Animal Science, National Chung Hsing University, Taichung 40227, Taiwan; eve.lumsangkul@gmail.com (C.L.); samchiang@nchu.edu.tw (H.-I.C.); 2 Department of Animal Science and Biotechnology, Tunghai University, Taichung 40704, Taiwan; nlo@thu.edu.tw; 3 Graduate Institute of Biomedical Sciences, China Medical University, Taichung 40402, Taiwan; 4 Translational Medicine Research Center, China Medical University Hospital, Taichung 40402, Taiwan; 5 Department of Bioinformatics and Medical Engineering, Asia University, Taichung 41354, Taiwan

**Keywords:** *Fumonisin* B_1_, developmental toxicity, embryogenesis, NTD, teratogen

## Abstract

A teratogenic agent or teratogen can disturb the development of an embryo or a fetus. *Fumonisin* B_1_ (FB_1_), produced by *Fusarium verticillioides* and *F. proliferatum*, is among the most commonly seen mycotoxins and contaminants from stale maize and other farm products. It may cause physical or functional defects in embryos or fetuses, if the pregnant animal is exposed to mycotoxin FB_1_. Due to its high similarity in chemical structure with lipid sphinganine (Sa) and sphingosine (So), the primary component of sphingolipids, FB_1_ plays a role in competitively inhibiting Sa and So, which are key enzymes in de novo ceramide synthase in the sphingolipid biosynthetic pathway. Therefore, it causes growth retardation and developmental abnormalities to the embryos of hamsters, rats, mice, and chickens. Moreover, maternal FB_1_ toxicity can be passed onto the embryo or fetus, leading to mortality. FB_1_ also disrupts folate metabolism via the high-affinity folate transporter that can then result in folate insufficiency. The deficiencies are closely linked to incidences of neural tube defects (NTDs) in mice or humans. The purpose of this review is to understand the toxicity and mechanisms of mycotoxin FB_1_ on the development of embryos or fetuses.

## 1. General Features of *Fumonisin* B_1_

Mycotoxin *Fumonisin*s (FBs) are fungal secondary metabolites produced by *Fusarium verticillioides* and *F. proliferatum*. They are water soluble and contain two propane-1, 2, 3-tricarboxylic acid side chains, esterified to an aminopolyol backbone. Eighteen different related types have been identified and isolated [1,2]. *Fumonisin* B_1_ (FB_1_) is clearly the most abundant analogue and the most prevalent mycotoxin contamination found in stale corn [3,4,5,6,7,8,9].

FB_1_ contamination occurs in maize kernels and biosynthesis of FB_1_ within the environment of the fungus-colonized kernel can be affected by the kernel composition. FB_1_ production is at least five times higher in de-germed maize kernels than in germ tissue [10]. FB_1_ biosynthesis varies with the developmental age of the tissue with the highest level of FB_1_ appearing in the later stages of kernel development [11].

More than 50% of FB_1_ contamination has been found in spoiled corn and corn-based products, in many parts of the world. A wild range of concentrations of FB_1_ from 6 to 155,000 μg/kg was detected in the investigated corn samples [12,13,14,15,16,17,18,19] that exceeded both the U.S. Food and Drug Administration guidelines and the EU maximum limits in de-germed dry-milled corn products (2000 μg/kg of total FB) [4,5,6,7,8]. In South America, all Brazilian corn meal samples were found to contain 1310 to 19,230 μg/kg of FBs [17]. Maize and maize-based foods, such as the cornflakes and corn snacks, have become an integral part of human life, being consumed on a daily basis. It has been shown that total maize production increased from 832.5 to 1099 million metric tons, globally, between 2011 and 2018 [20,21]. Similarly, total corn consumption around the world summarized by USDA increased from 991 to 1131 thousand metric tons, between 2015 and 2018 [22].

According to WHO (2001), the maximum tolerable daily intake of FBs, for humans, is 2 μg/kg-BW (body weight) [23]. The European Commission (2006 and 2007) also established a maximal FB level of 1000 μg/kg in maize and maize-based food for humans, 800 μg/kg in maize-based breakfast cereals and snacks, and 200 μg/kg in maize-based infant food [24,25]. Therefore, children and infants are the main risk groups for FB_1_ toxicity. In Brazil, Tanzania, Guatemala, South Africa, and Argentina [26,27], an assessment revealed that human consumption of FB_1_ is above the tolerable daily level. Prevalence of esophageal cancer in Africa and Asia is also the highest in areas with high concentrations of FB_1_ contamination reported (between 140,480 and 155,000 μg/kg) [18,19]. As corn is also one of the primary components of animal feeds, animals are also among those at a high risk of FB_1_ contamination. 

It has been reported that FB_1_ induces many animal diseases, such as equine leukoencephalomalacia [28], porcine pulmonary edema syndrome [29], hepatic tumor in rats [30], acute and fatal nephrotoxicity and hepatotoxicity in lambs [31]. Various degrees of toxic responses have been observed in chickens, ducklings, and turkey poults (e.g., decreased body weight gain, increased mortality, reduced size of the bursa of *Fabricius*, thymus, and spleen, myocardial degeneration, myocardial hemorrhage, alterations in the hemostatic mechanism and necrosis of hepatocytes) [32,33,34,35,36,37]. Therefore, mycotoxins not only pose a significant risk to human and animal health, but also impact food security and reduce livestock production. Doubtless, FB_1_ has been one of the most hazardous mycotoxins with regard to animal health that is tightly associated with economic losses. 

## 2. Toxicokinetic of FB_1_

Mycotoxin FB_1_ is difficult to absorb and can be rapidly excreted (urinary and biliary) in most species [38]. In rats, FB_1_ can be excreted with feces (80%) and urine (3%), 96 h after intragastric administration of radiolabeled [^14^C] FB_1_. However, low but consistent FB_1_ levels can still be detected in the liver, kidneys, and blood [39]. Although the liver and kidneys are two major target organs retaining most of the absorbed toxins, FB_1_ is also found in serum and other tissues in pigs, after oral administration [40,41]. 

Intestinal absorption of FB_1_ and its biliary excretion are involved in the potential interactions between FB_1_ and cholesterol [42]. Through interactions with cholesterol and bile salts, such as sodium taurocholate, dietary FB_1_ could be incorporated into mixed micelles. Transfer of FB_1_ to micelles facilitates its intestinal absorption [42]. A few studies have looked at the transfer of FB_1_ from intestine to uterus or eggs. Previous studies in mammalian species suggested that FB_1_ could cross the placenta when the pregnant mouse was exposed to FB_1_, during the early gestation period (embryonic day 7.5–8.5, E7.5–8.5) [43]. In the early gestation stage, the placenta is not yet well-formed, which potentially leaves the embryos vulnerable to teratogenic insults. However, when pregnant rats were exposed to FB_1_ at later gestation period (E15), embryo development was not affected by the toxins, suggesting that the fully developed placenta might provide a protective barrier against a mycotoxin attack [44]. Nevertheless, no evidence has shown such FB_1_-placenta or FB_1_-egg transportation in humans and in avian species.

The FB_1_ molecule includes a long chain aminopentol backbone (AP_1_) with two ester-linked tricarballylic acids (TCA), in which AP_1_ originates from the hydrolysis of the tricarballylic acid side chains, at carbons 14 and 15; it is then replaced by the hydroxyl groups [45]. Recent studies have demonstrated that swine cecal microbiota can metabolize FB_1_ to partially hydrolysed FB_1_ (PHFB_1_) and a small amount of AP_1_ [46]. In weaning piglets, the presence of PHB_1_ and AP_1_ is found in tissues with mostly unmetabolized FB_1_ [47].

Based on the intraperitoneal or intravenous dosing, the kinetics of FB_1_ elimination is consistent with the one or two-compartment model. Depending on animal species, the initial elimination of FB_1_ is rapid with a half-life of approximately 10 to 116 min, among different animals [48,49,50,51,52,53]. In weaning piglets, however, FB_1_, PHB_1_, and AP_1_ were detected in animal tissues, during a ten-day long elimination period [47].

## 3. Mechanisms through Which the FB_1_ Exerts Its Developmental Toxicity 

Teratogens are known to interfere with embryonic or fetal development and cause congenital malformations (birth defects). Potential teratogens include radiation, maternal infections, chemicals and drugs, etc. [54]. In the case of corn contaminated with FB_1_, physical or functional defects in human and animal embryos or fetuses are possible when pregnant mothers are exposed. Therefore, FB_1_ has also been proven to be among those potential teratogens during embryonic development.

**Figure 1:**
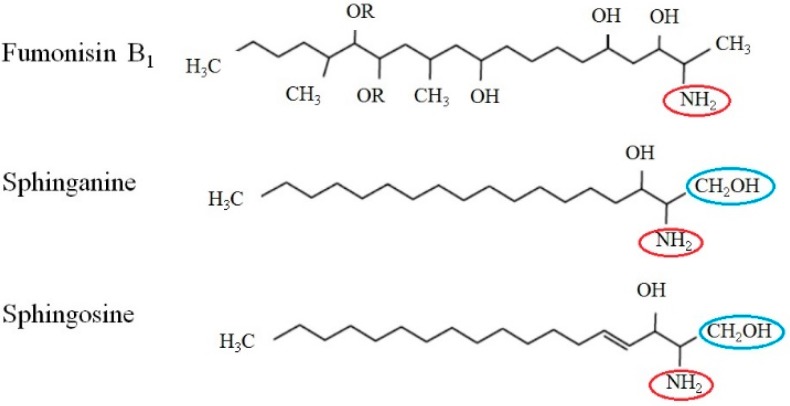
Comparison of chemical structure of FB_1_ and primary components of sphingolipids, sphinganine (Sa) and sphingosine (So). The chemical structure of FB_1_ has a high similarity to Sa and So, which all possess an amine group (red circle) attached to the long fatty-acid chain. FB_1_ also differs from Sa or So, by the absence of a hydroxymethyl group (blue circle) attached to the head group [1,2,3,55].

**Figure 2:**
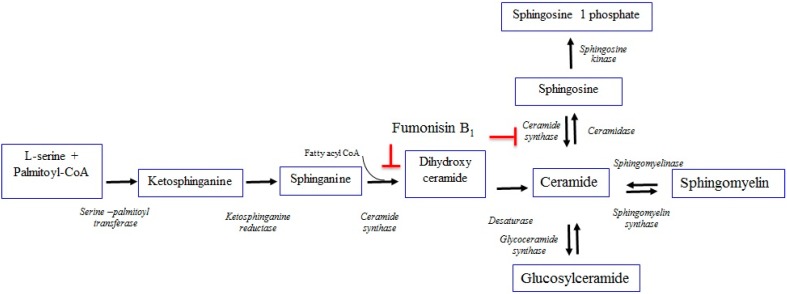
Inhibition of the ceramide synthesis pathway by FB_1_. In normal physiology, the amino group of Sphinganine (Sa) and Sphingosine (So) can form an amide bond with fatty acid carboxyl to produce a ceramide. Mycotoxin FB_1_ can bind to the catalytic site of ceramide synthase, resulting in an inhibition of the reduction of Sa with fatty acyl CoA forming dihydroceramide. Furthermore, re-acylation of So, derived from breaking down sphingolipids to form ceramide, is also inhibited by FB_1_ (red line) [56,59,60,61,62,63,75].

The best-known mechanism of action for FB_1_ is closely associated with sphingolipid metabolisms. Due to the similarity of its chemical structure (Figure 1) with Sphinganine (Sa) and sphingosine (So) [1,2,3,55], FB_1_ can interfere with the metabolism of Sa and So, the primary components of sphingolipids. Sphingolipids are widely distributed compounds and are often part of biomembranes. They regulate critical cell functions, such as cell proliferation, differentiation, and apoptosis [56,57]. Therefore, during exposure, FB_1_ serves as a competitive inhibitor for the Sa and So key enzymes in the de novo synthesis of ceramide, complicating the sphingolipid biosynthesis pathway (Figure 2) [58,59,60]. Inhibition of the ceramide synthase leads to a rapid increase of sphinganine, reduced sphingosine, elevated Sa/So, accumulations of the 1-phosphate metabolites of Sa/So, as well as the decreased downstream sphingolipids [61,62,63].

Disturbance in the sphingolipid metabolism is thought to be a major contributor to FB_1_ toxicity; it can also interfere with folate transporters. In other words, another developmental toxicity of FB_1_ is disruption of folate processing, due to its high-affinity for the folate transporter. This, in turn, can lead to an insufficient transfer of folates to a developing fetus, causing folate-deficient syndromes, during embryogenesis. This phenomenon is similar to that observed when pregnant animals are fed specific vitamin-deficient diets, such as folate-deficient diets. Therefore, FB_1_ inhibition of ceramide biosynthesis can be plausibly linked to folate insufficiency and is closely associated with an increased risk of neural tube defects (NTDs) [64,65,66,67,68,69]. 

A possible etiological factor for embryo deaths and fetal malformations is maternal toxicity [70,71,72]. Collins et al. suggested that FB_1_ was not teratogenic but that developmental variations induced by the mycotoxin were secondary to maternal toxicity. Overall, FB_1_ exerts secondary to maternal toxicity, which includes growth impairment at high doses and fetal death in utero. In this study, signs of maternal toxicity (increased Sa/So ratios characteristic in maternal livers, kidneys, serum, and brains) were observed in kidneys and livers in all FB_1_-treated groups, but with no effect in fetuses [73]. Similarly, Reddy et al. found FB_1_-treated mice had increased Sa/So ratios in maternal organs, but not in fetal livers [74]. Therefore, the effects of FB_1_ on fetuses, should be considered as secondary to maternal toxicity.

## 4. *Fumonisin* B_1_ and Developmental Toxicity in Animals

As mentioned previously, mycotoxin FB_1_ might act as an embryonic or fetal cytotoxic agent (secondary to maternal toxicity), which results in growth retardation and developmental abnormalities, and indirectly induces NTDs when administered to pregnant animals. Previous studies about FB_1_ on developmental toxicity in rats, Syrian hamsters, mice, rabbits, humans, ruminants, and chickens are reviewed and summarized in Table 1.

### 4.1. Mammalian Models

#### 4.1.1. Rats

In vitro studies have shown that FB_1_ retarded the growth of cultured rat embryos exposed to FB_1_ concentrations ≥0.2 ppm, for 45 h, starting from E9.5. Phenotypic abnormalities significantly increased when FB_1_ concentrations were greater than 0.5 ppm and reached 100% prevalence at 1.01–40.4 ppm [44]. Similarly, in rat embryos cultured with aminopentol (AP_1_; 0, 2.17, 7.22, 21.7, 72.2, or 217 ppm), the hydrolysis product of FB_1,_ a significant increase was observed in the incidence of abnormal embryos (NTDs and other abnormalities) [76]. In both studies, the observed abnormalities were mainly in conjunction with the retardation of overall growth and development, suggesting that both AP_1_ and FB_1_ uniformly exert embryo-toxicity, in detriment to the entire embryo.

In contrast, results from an in vivo study found no overt adverse effects on the reproductive performance of either male or female rats, or their offspring when the rats were fed with FB_1_ (1, 10, or 55 ppm), starting 9 and 2 weeks before mating. Briefly, litter weight gains in the 10 and 55 ppm FB_1_ groups were slightly decreased; however, gross litter weight and physical development of offspring were not affected. Furthermore, altered sphingolipid ratios, specifically increased sphinganine to sphingosine ratios, were found in the livers of dams from the highest (55 ppm) FB_1_ treatment group. However, sphingolipid ratios of abdominal sections, containing livers and kidneys, of fetuses showed no differences between the control and the highest dose litters [77].

#### 4.1.2. Hamsters

In Syrian hamsters, fetal body weight and crown-rump length appeared to decrease with the increased fetal deaths observed in dams, when the hamsters were given an oral administration of 6, 12, or 18 mg/kg-BW of FB_1_ on E8 and E9, respectively [78]. A similar result was also reported in pregnant Syrian hamsters that were given 0, 8.7, 10.4, 12.5, 15, or 18 mg/kg-BW of FB_1_ by gavage, on E8 through E12. Their litter sizes were significantly reduced when fed with higher doses of FB_1_ at 15 or 18 mg/kg-BW [79].

#### 4.1.3. Mice

Cultured mouse E9 embryos were exposed to a long-term (0, 0.72, 1.44, 2.16, 3.60, 5.05, 10.8, 18.0, 36.0, or 72.1 ppm FB_1_, for 26 h) or a short-term (36 ppm FB_1_ for 2 h) FB_1_ treatment. For 26 h of exposure, FB_1_ caused growth retardation and malformations of embryos, at all concentrations equal to or greater than 1.44 ppm. For instance, one of the NTD phenotypes (exencephaly) was observed at concentrations ≥1.44 ppm of FB_1_ treatments, and its incidence increased from 10% at 1.44 ppm to 48% at 72.1 ppm (the highest concentration). At a 2 h exposure with 36 ppm FB_1_, the mouse embryo showed a retarded growth, with NTDs and facial hypoplasia observed in 67% and 83% of the tested embryos, respectively, [80]. 

When inbred LM/Bc mice were given intraperitoneal dosages of FB_1_ (0, 5, 10, 15, or 20 mg/kg) on E7.5 and E8.5, the incidence of fetuses showing exencephaly increased from 5% at 5 mg/kg to 79% at 20 mg/kg-BW of FB_1_ treatments [43]. Similarly, Voss et al. found the incidence of NTD litters was 4/11 (36%), at 10 mg/kg-BW (maternal) of the mycotoxin given (intraperitoneal injection) to LM/Bc mice on E7 and E8 [64].

Other experiments have indicated that CD1 mice are more resistant to NTDs induced by FB_1_ than the LM/Bc mice. Tests on CD1 mice have shown that the fetal toxicity (fetal death and decreased fetal weight) was evident after exposure to FB_1_ [74]. Voss et al. reported that developmental NTDs were less than 10% when mice were given 10 mg/kg-BW of FB_1_ [81]; however, FB_1_ was found to be fetotoxic, at a maternal dose ≥45 mg/kg-BW, where 36% of mouse fetuses with NTDs were induced. Moreover, Liao et al. also showed that CD1 mice fed with 12.5 mg/kg had NTDs in 7.4% of the mouse fetuses examined [82].

The comparison between the LM/Bc and CD1 females by Voss et al. [83] revealed a 10% incidence (1/10 fetuses) of exencephaly in the LM/Bc mouse fetuses, when female mice were fed with diets contaminated with 150 ppm FB_1_; but no NTDs were found in the CD1 offspring, although the CD1 litters exhibited a higher fetal mortality. Conceivably, differential responses or sensitivity to FB_1_ treatment could also exist even between these two mouse strains and not only among different animal species.

#### 4.1.4. Rabbits

When given to New Zealand White rabbits by gavage (0, 0.1, 0.5, or 1.0 mg/kg-BW on E3 through E19 embryos), FB_1_ caused maternal mortality and disrupted maternal sphingolipid metabolism at concentrations ≥0.5 mg/kg-BW. Apparently, FB_1_ is fetotoxic at the two highest doses of 0.5 and 1.0 mg/kg-BW, as indicated by the reduced fetal body, kidney, and liver weights [84]. Exposure of male rabbits to FB_1_ contaminated diets with up to 7.5 mg/kg FB_1_, depressed the testicular and epididymal sperm reserves, the sperm production, and potentially impaired reproduction of the buck, and in turn, induced embryo mortality, during later development [85]. Evidence of disrupted sphingolipid metabolism was not found in rabbit fetuses, suggesting that the effect of FB_1_ on rabbits were also secondary to maternal toxicity.

#### 4.1.5. Humans

Although FB_1_ has been identified with significant health threats in livestock and many other animals, evidence for the same in humans is currently inconclusive. Some studies expressed concerns that exposure to FB_1_ might contribute to serious adverse health outcomes, such as cancers and birth defects. One study on the incidence of NTDs among Mexican Americans at the Texas–Mexico border, suggested that FB_1_ exposure in pregnant women might be a contributing factor to NTDs in babies. The NTD risk can increase in women consuming homemade tortillas, which contain approximately 0.234 ppm FB_1_, with daily exposure, approximately 0.1726 mg/day/kg-BW compared to the control group [91]. Callihan et al. suggested that the mechanism of FB_1_ action has been confirmed in humans [86]. The alteration of sphingosine 1-phosphate (S1P) and its receptors, during the development of the nervous system, were observed. For instance, human neuroepithelial cells (hES-NEP) treated with various concentrations of (0.00072, 0.0072, 0.072, 0.72, 7.2, or 72 ppm) FB_1_, for 48 h, caused the inhibition of ceramide synthases and resulted in an accumulation of sphingoid-based dihydrosphingosine and the bioactive lysophosphosphingolipid dihydro-sphingosine 1-phosphate (dhS1P).

#### 4.1.6. Cattle

The study of FB_1_ on developmental toxicity in ruminants is relatively limited, compared to those in other animal species and humans. However, in vitro oocyte maturation (IVM) was compromised by FB_1_ treatments, when bovine cumulus-oocyte complexes (COCs) were matured for 22 h, in a maturation medium containing 3.6, 7.2, 14.4, 21.6, or 36.0 ppm of FB_1_. Oocytes matured in the medium with 7.2 ppm FB_1_, decreased the proportion of the two- to four-celled embryos; oocytes matured with 14.4 ppm FB_1_ had no changes in their cleavage rates but showed a reduced blastocyst rate on day 8, post-fertilization. When the highest concentration (36.0 ppm) of FB_1_ was used, the most prominent effect on oocyte development was observed, in which the oocytes could not be normally fertilized or developed to the blastocyst stage [87]. In general, bovine oocytes matured in FB_1_-containing medium, also compromised the quality of developing embryos.

### 4.2. Avian Species

Javed et al. inoculated chicken eggs with (0, 0.72, 7.2, or 72 ppm) FB_1_ or *Fusarium proliferatum* culture material extract (CME), to provide 14.4 ppm of FB_1_ and 2.82 ppm FB_2_, on day 1 or day 10 of the 21-day incubation period. They found FB_1_ increased embryo mortality from 50% to 100%, when inoculated with FB_1_, compared to a 100% mortality in the CME treatment. Early fetal abnormalities including hydrocephalus, enlarged beaks and elongated necks, were also observed in FB_1_-exposed embryos; pathologic changes were evident in livers, kidneys, heart, lungs, musculoskeletal system, intestines, testes, and brains, in these toxin-exposed embryos [88]. In agreement with Bacon et al., a significantly increased mortality of embryos was observed in the FB_1_-administered group [89]. Another study was performed by Henry et al. to confirm FB_1_ toxicity, where broiler embryos were injected with 0 to 0.25 ppm FB_1_, followed by 72 h of incubation. By day 18, after FB_1_ injection, the cumulative embryonic mortality (56%) drastically increased, compared to the control group (4%) [90]. It is, hence, clearly demonstrated that exposure to mycotoxin FB_1_ adversely affected embryo survival and development in poultry. Unlike mammalian species, however, it remains unclear whether maternal exposure to mycotoxin FB_1_ (acute and chronic) can cause accumulative effects that could directly carry over to the developing chick embryos. It would be of great interest to develop more in-depth studies to reveal this maternal–fetal portal of *Fumonisin* toxicity.

## 5. Concluding Remark

Mycotoxin FB_1_ apparently acts directly or indirectly as an embryotoxic or fetotoxic teratogen to cause growth retardation, delayed or incomplete organogenesis, malformations, and ultimately fetal death, in several species, largely in a dose-dependent manner. Based on histopathological and sphingolipid profile assessments of the dams, fetotoxicity secondary to maternal toxic effects are also prominent. The mechanism of action for the toxicity of mycotoxin FB_1_ is understood to be through the competitive inhibitors of ceramide synthase in the de novo sphingolipid biosynthetic pathway. However, there is still no adequate evidence to implicate the initial alterations caused by FB_1_, particularly during early embryogenesis; similar studies in domestic species are also largely unavailable. Nevertheless, comparative studies between sensitive and non-sensitive animals might be necessary to determine which animal model is most relevant to humans. It would be useful to further investigate the specific toxicity and derive more conclusive mechanisms in *Homo sapiens.* Furthermore, due to the frequent higher level of FB_1_ contaminants, compared to the safety allowance recommended by the European Union (Commission of European Communities) and the USFDA Center for Food Safety and Nutrition, it is essential to develop an effective strategy to minimize risks of FB_1_ exposure for both animal species and human beings.

## Figures and Tables

**Table 1:** The developmental toxicity of mycotoxin *Fumonisin* B_1_ in animals and humans.

Species	FB_1_ Treatment and Doses	Developmental Toxicity	Reference
Rat (Sprague-Dawley)	Rat embryos cultured in 0.2 to 40.4 ppm FB_1_ for 45 h at embryonic day (E) 9.5	Retarded development; increased abnormal embryos phenotype	[44]
Rat (Sprague-Dawley)	Rat embryos cultured in 0, 2.17, 7.22, 21.7, 72.2 or 217 ppm hydrolized FB_1_ (HFB_1_) for 45 h at E9.5	Increased percentage of NTDs and other abnormalities	[76]
Rat (Sprague-Dawley)	Male and female rats fed 0, 1, 10, or 55 ppm FB_1_ 9 or 2 wk prior to mating	No effect on the peformance of dam or embryos; maternal toxicity by altering sphingolipid metabolism in the livers of dam at the highest dose	[77]
Syrian Hamster	Female hamsters fed 0, 6, 12, 18 mg/kg-BW by gavage on E8–E9	Increased fetal death; malformation at the highest dose	[78]
Syrian Hamster	Pregnant Syrian hamsters given 0, 8.7, 10.4, 12.5, 15, or 18 mg/kg-BW by gavage on E8–E12.	Reduced litter size; decreased fetal weight; reduced body length	[79]
Mouse (ICR)	Mouse embryos cultured with 0, 0.72, 1.44, 2.16, 3.60, 5.05, 10.8, 18.0, 36.0 or 72.1 ppm for 26 h and 36 ppm for 2 h	Induced facial NTDs; growth retardation	[80]
Mouse (LM/Bc)	Pregnant mice injected with 0, 5, 10, 15 and 20 mg/kg-BW/day via intraperitoneal on E7.5 and E8.5	Increased exencephaly from 5% to 79%	[44]
Mouse (LM/Bc)	Female mice treated with 0, 2.5 or 10 mg/kg by intraperitoneal on E7 and E8	NTDs (36%) in 10 mg/kg treated group	[64]
Mouse (CD1)	Female mice fed 0, 12.5, 25, 50 and 100 mg FB_1_/kg-BW by gavage from E7–E15	Fetal toxicity; increased fetal death; decreased fetal weight	[74]
Mouse (CD1)	Pregnant mice injected with 15, 30 or 45 mg FB_1_/kg-BW/day (1st trial); 10, 23, 45 or 100 mg/kg-BW/day (2nd trial) via intraperitoneal at E7 and E8	Fetotoxicity; increased NTDs (8–55%)	[81]
Mouse (CD1)	Pregnant mice fed 12.5 mg/kg FB_1_ at E7.5 and E8.5	NTDs (7.4% exencephaly)	[82]
Mouse (LM/Bc, CD1)	Female mice fed 0, 50 or 150 mg/kg diet at wk 5 before mating and after mating until E16	10% of exencephaly in LM/Bc mice (at 150 mg/kg); No NTDs but fetal death in CD1 mice	[83]
Rabbits (New Zealand White)	Rabbits fed 0, 0.1, 0.5, and 1.0 mg/kg-BW by gavage during E3–E19	Decreased body and organ weights of fetuses	[84]
Rabbits (NewZealand White x Chinchilla)	Male rabbits fed 0.13, 5.0, 7.5 and 10 mg/kg for 28 wk then mated with female rabbits	Induced embryo mortality	[85]
Human neural epithelial cells	Cells treated with 0.00072, 0.0072, 0.072, 0.72, 7.2, and 72 ppm FB_1_ for 48 h	Altered balance of Sphingosine 1-phosphate	[86]
Bovine oocytes	Cumulus-oocyte complexes matured in 3.6, 7.2, 14.4, 21.6, or 36.0 ppm FB_1_-containing medium for 22 h	Decreased percentages and quality of matured oocytes and embryos	[87]
Chicken (Columbia x New Hampshire)	Eggs inoculated with 0.72, 7.2 or 72 ppm of FB_1_ or 14.4 ppm FB_1_ + 2.82 ppm FB_2_, and 0.84 ppm moniliformin at the air sac on day 1 or day 10 of incubation	Increased mortality rates; altered brain, beak, and neck development; pathologic changes in livers, kidneys, heart, lungs, musculoskeletal system, intestines, testes, and brains	[88]
Chicken embryos (White Leghorn)	Chicken embryos injected with 0, 0.017, 0.085, 0.17, 0.425, 0.85, 1.275 and 1.74 ppm FB_1_ at day 1 of incubation	Increased embryonic death	[89]
Chicken embryos (Peterson-Arbor Acres)	Chicken embryos injected with 0 or 0.25 ppm FB_1_ at 72 h of incubation	Increased embryonic mortality on d 18	[90]
